# Prevalence and factors associated with malnutrition among under 5-year-old children hospitalised in three public hospitals in South Africa

**DOI:** 10.4102/phcfm.v12i1.2444

**Published:** 2020-11-27

**Authors:** Makanda B. Itaka, Olufemi B. Omole

**Affiliations:** 1 Division of Family Medicine, Faculty of Health Sciences, University of the Witwatersrand, Johannesburg, South Africa

**Keywords:** factors, malnutrition, under 5 years, hospitalised, children

## Abstract

**Background:**

Malnutrition is a significant risk factor for ill health among children under 5 years of age and the consequences are significant.

**Aim:**

The aim of this study was to determine the prevalence and factors associated with malnutrition among under-5-year-old hospitalised children.

**Setting:**

This study was set at Sebokeng, Kopanong and Heidelberg hospitals, Sedibeng district, South Africa.

**Methods:**

This was a cross-sectional study comprising 306 hospitalised under-5-year-old children. Information on socio-demography, feeding practices, immunisation and clinical problems was obtained from caregivers and medical records. Anthropometric measurements were also performed.

**Results:**

Most participants were male (59.8%), had normal birth weights (80.0%), come from a household with a monthly income R2000 (about 150 US dollars) (50.3%), up-to-date immunisation (97.4%), breastfed for 6 months (57.4%) and were fed 3–4 meals/day (66.7%) and, at most, one snack/day (63.4%). Acute malnutrition accounted for 9.5% (*n* = 29) of admissions. Among these, 82.8% (*n* = 24) had severe acute malnutrition. On test of association, monthly household income (*p* = 0.01), mother’s and father’s employment status (*p* = 0.01; *p* = 0.01), breastfeeding history (*p* = 0.01) and having diarrhoea in index admission (*p* = 0.01) were significantly associated with malnutrition admission. In multivariate regression analyses, not being breastfed (odds ratio [OR] = 3.9; 95% confidence interval [CI]: 1.23–12.29; *p* = 0.02) and diarrhoea at index admission were independently associated with malnutrition (OR = 23.3; 95% CI: 6.85–79.43; *p* = 0.01).

**Conclusion:**

A significant proportion of participants had malnutrition and were subjected to suboptimal feeding practices. Healthcare providers in primary care need to entrench dietary education and anthropometric screening in all clinic visits for children 5 years old, particularly when they present with diarrhoea or are not being breastfed.

## Introduction

Malnutrition remains a significant risk factor for ill-health among under 5-year-old children, and the short- and long-term consequences are significant.^1^ Although substantial progress has been made globally in reducing the number of deaths among children in this age group,^2^ one child out of every 13 in sub-Saharan Africa still dies before their fifth birthday, from preventable health problems, including malnutrition.^2,3^ South Africa Demographic and Health Survey 2016 reported that 27.0% of under 5-year-old children were stunted in South Africa.^4^

The risk of malnutrition in the under 5-year-old age group is related to the increased nutritional needs that are indispensable for the growth of young children.^5^ The World Health Organisation (WHO) therefore refers to childhood malnutrition as the most lethal form of malnutrition because this predisposes them to serious health, cognitive and developmental problems, reduced quality of life and even death.^6^ Factors that have been implicated in the aetiogenesis of childhood malnutrition include insufficient diet or calorie intake, frequent infections, sociocultural taboos that exclude certain food elements, poor psychosocial care, neglect, abnormal mealtimes and financial problems.^5^

In South Africa, several studies have examined the prevalence, associated factors, impact and the double burden of malnutrition (underweight and overweight).^7^ Most of these studies are focussed on the rural areas with very few conducted in the urban and peri-urban settings. However, the geographical landscape typical of South Africa means that peri-urban informal settlements also experience socio-economic deprivations, and sometimes even worse than in rural areas. While Gauteng province is the economic hub of South Africa, there are several pockets of socio-economically deprived communities within this success story. Of the five health districts in the Gauteng province, Sedibeng is the most economically challenged with several communities typical of this socio-economic deprivation.^8^ Data from the district health information system (DHIS) in 2015 and 2016 indicate a very high in-hospital case fatality rate of 13.8% among children younger than 5 years, a significant proportion of which is because of severe acute malnutrition (SAM).^9^ Such high fatality rate raises serious clinical and public health concerns and makes it imperative to explore factors that may be influencing malnutrition in this district. The aim of this study was therefore to determine the prevalence and factors associated with malnutrition among under 5-year-old children admitted to the three public hospitals in Sedibeng District, South Africa.

## Methods

### Design

This was a cross-sectional study with analytical component.

### Setting

This study was conducted at the paediatric wards of Heidelberg, Kopanong and Sebokeng hospitals. The first two are district hospitals with 126 and 248 beds, respectively, while Sebokeng is a regional specialist hospital with 800 beds.

### Participants and sampling

All children aged 1–59 months admitted to the paediatric wards of the three hospitals from 15 March to 15 May 2016 were eligible for the study. During 2014 and 2015, 153 of 1463 (10.5%) of the children aged 1–60 months admitted to the three hospitals were for malnutrition.^9^ Assuming a confidence interval (CI) of 95%, a sampling error of 5% and a response distribution of 50%, the minimum sample required for this study, as determined using Raosoft sample size calculator,^10^ was 306. This sample was proportionally divided among the three hospitals, based on their contribution in 2014 and 2015 to the total number of admissions. This resulted in 78 children being sampled from Heidelberg Hospital, 81 from Kopanong Hospital and 148 from Sebokeng Hospital.

Participants were consecutively recruited during admission. Caregivers were approached and briefed by the ward clerk or research assistant on the nature and purpose of the research. Each carer was invited to participate, and assent and consent obtained from parents or legal guardians. If a caregiver did not want to participate, the next caregiver was approached, and recruitment continued until sample size was reached over a period of 2 months. Children who were too sick to participate and those admitted to the intensive care unit were excluded.

### Measuring tools and data collection

Prior to proceeding with data collection, the first author trained the research assistant and ward clerk on the aim and objectives of the study, and how to administer the questionnaire independently. Where the participants could not speak English, the research assistant or ward clerk interpreted for the researcher.

A researcher-administered questionnaire adapted from a previous study^11^ was used for data collection, after permission was obtained from the authors. The following sections of the questionnaire were adapted to address the aim and objectives of this study:

- The question on religion listed under caregiver’s characteristics was removed.
- The questions relating to the source and quality of water, dietary practices, diarrhoea, health characteristics and immunisation were adapted to local contexts.

Caregivers were approached by the ward clerk as part of his normal duties of obtaining information from patients and capturing this electronically. After the consent form was signed, the questionnaire was administered by the research assistant alone or in the presence of the researcher. Interviews were conducted within 2 days of admission to avoid significant changes in anthropometric parameters. Each interview lasted a maximum of 30 min.

Data collected included information on each participant’s demographic profile, household, socio-economic status and medical conditions. Additional information on socio-economic status, clinical condition, environmental condition, breastfeeding and dietary practices was extracted from the child’s medical records. Anthropometric parameters (weight, height or length and mid-upper arm circumference [MUAC]) were measured by the first author. Other information such as immunisation and deworming status and previous episodes of clinic attendance for illness were collected from the Road-to-Health Card (RTHC).

Each participant was given a unique code. Data from all completed questionnaires were captured into a secured computerised file. Only the research team had access to this information.

## Data analysis

Data were captured onto Microsoft Excel spreadsheets and imported into STATA statistical analysis software, version 14.0, with the assistance of a statistician. Descriptive statistics were used to summarise participants’ socio-demographic, clinical, dietary and anthropometric characteristics. These were reported as frequencies, percentages (categorical data) and means with standard deviations (continuous data). Where data were not deemed normally distributed, medians and interquartile ranges were reported.

The proportions of participating children with different categories of malnutrition were determined based on the WHO classification. Moderate acute malnutrition (MAM) was defined as a standard deviation of less than –2 for height-for-age (stunting) or for weight-for-age (underweight). Severe acute malnutrition was defined as a standard deviation of less than –3 for weight-for-height.

The sample was divided into two groups: those who were wellnourished and those who were malnourished (MAM or SAM). These were compared by using Pearson’s Chi-square test and logistic regression in terms of socio-economic, dietary and clinical characteristics. Where statistically significant associations were detected, further analyses were carried out by using multivariate logistic regression to determine factors independently associated with malnutrition. Statistical significance was taken as *p* < 0.05.

### Ethical consideration

Ethics clearance was obtained from the Human Research and Ethics Committee of the University of the Witwatersrand (Number M161041). Permission was obtained from the Sedibeng District Health Services management. To ensure anonymity, questionnaires were coded and did not collect personal identifiable data.

## Results

Three hundred and six children participated in the study. Participants were almost equally distributed between the provincial regional (48.4%, *n* = 148) and district hospitals (51.6%, *n* = 158) (see Table 1).

**TABLE 1:** Number of participants admitted with malnutrition per hospital.

Hospital	MAM	SAM	Total (MAM and SAM)
Sebokeng	4	10	14
Kopanong	1	7	8
Heidelberg	0	7	7

**Total**	5	24	29

MAM, moderate acute malnutrition; SAM, severe acute malnutrition.

As shown in Table 2, most participants were male (59.8%), > 12 months of age (52.9%), had normal birth weights (80.7%), had a total household income of < R2000 per month (50.3%) and were up to date with immunisation (97.4%). Pneumonia (52.3%) and diarrhoea (20.9%) were the two most common presenting medical problems.

**TABLE 2:** Socio-demographic and dietary characteristics of the children (*N* = 306).

Characteristics	*n*	%	Median	IQR
**Age (months)**	-	-	14	6–29
< 6	74	24.2	-	-
6–12	70	22.9	-	-
> 12	162	52.9	-	-
**Gender**	-	-	-	-
Male	183	59.8	-	-
Female	123	40.2	-	-
**Birth weight (g)**	-	-	2905	2600–3200
Birth weight 2500 or more	247	80.7	-	-
Low birth weight < 2500	43	14.1	-	-
Very low birth weight < 1500	15	4.9	-	-
Extremely low birth weight < 1000	1	0.3	-	-
**Children ever been breastfed**	-	-	-	-
No	88	29.8	-	-
Yes	207	70.2	-	-
**Duration of breastfeeding (months)**	-	-	6	6–9
< 6	87	42.0	-	-
6 or more	120	58.0	-	-
**Mothers currently breastfeeding**	-	-	-	-
*No*	220	74.6	-	-
Yes	75	25.4	-	-
**Reasons for stopping breastfeed**	-	-	-	-
Age of the child	34	15.4	-	-
Refused	31	14.0	-	-
Breast problem	58	26.2	-	-
Sick	71	32.1	-	-
School or work	27	12.2	-	-
**Children started other food beside breastfeeding**	-	-	-	-
No	76	24.8	-	-
Yes	230	75.2	-	-
**Age of starting other food (months)**	-	-	6	6–9
Less than 6	44	19.1	-	-
6–9	170	73.6	-	-
More than 9	17	7.4	-	-
**Number of meals per day**	-	-	4	3–5
Two meals per day	6	2.0	-	-
Three meals per day	132	43.1	-	-
Four meals per day	72	23.6	-	-
Five meals per day	29	9.5	-	-
Six meals per day	38	12.4	-	-
Seven meals per day	5	1.6	-	-
Eight meals per day	19	6.2	-	-
More than eight	5	1.6	-	-
**Number of snacks per day**	-	-	1	0–2
None	107	35.0	-	-
One snack per day	87	28.4	-	-
Two snacks per day	67	21.9	-	-
Three snacks per day	32	10.5	-	-
Four snacks per day	12	3.9	-	-
Five snacks per day	1	0.3	-	-

IQR, interquartile range.

Note: ‘*N*’ not always equal to 306 because of missing data.

In terms of diet, most participants were reportedly breastfed for > 6 months of age (57.4%, *n* = 120). However, at the time of the study, most were no longer breastfeeding (74.6%) and many had stopped because the child was sick (32.2%, *n* = 71) or there were breast problems in the mother (26.2%, *n* = 58). Although supplementary foods were started for most participants between 6 and 9 months of age (73.6%), one in five (19.1%) were on supplementary feeds before the age of 6 months. Most participants (66.7%) were fed only three to four meals a day and had at most one additional snack per day (63.4%).

Most carers were the mother of the baby (92.5%, *n* = 283) and were aged 18–35 years (84.0%), single (66.0%) and unemployed (71.2%). In contrast, most of the fathers were employed (60.1%). Most households had four or less dependents (77.5%), private water source (69.9%) and total≈monthly income of 2000 Rands ($150) or less (50.3%) (Table 3).

**TABLE 3:** Socio-demographic characteristics of parents or caregivers.

Characteristics	*n*	%	Mean	s.d.	Median	IQR
**Mother’s age (years)**	-	-	29	9	-	-
Less than 18	4	1.3	-	-	-	-
18–35	257	84.0	-	-	-	-
> 35	45	14.7	-	-	-	-
**Mother’s marital status**	-	-	-	-	-	-
Single	202	66.0	-	-	-	-
Married or living together	100	32.7	-	-	-	-
Others (divorced, widowed)	4	1.3	-	-	-	-
**Mother’s employment status**	-	-	-	-	-	-
Unemployed	218	71.2	-	-	-	-
Employed	88	28.8	-	-	-	-
**Father’s employment status**	-	-	-	-	-	-
Employed	122	39.9	-	-	-	-
Unemployed	184	60.1	-	-	-	-
**Household income category (Rands)**	-	-	-	-	2000	3000–4000
Less or equal to 2000	154	50.3	-	-	-	-
More than 2000	152	49.7	-	-	-	-
**Number of dependants**	-	-	-	-	3	2–4
Four or less dependants	237	77.5	-	-	-	-
More than four dependants	69	22.5	-	-	-	-
**Source of water**	-	-	-	-	-	-
Public tap	50	16.3	-	-	-	-
Private	214	69.9	-	-	-	-
Open in the yard	42	13.7	-	-	-	-
**Number of children < 5 years**	-	-	-	-	1	1–1
***Caregiver’s relationship with the child***	-	-	-	-	-	-
Mother	283	92.5	-	-	-	-
Grandmother	6	2.0	-	-	-	-
Caregiver	7	2.2	-	-	-	-
Father	10	3.3	-	-	-	-

s.d., standard deviation; IQR, interquartile range.

The median weight, height and MUAC of the participants were 9.15 kilograms (kg) (interquartile range [IQR]: 6.4–11.8), 75 centimetres (cm) (IQR: 61–88) and 14 cm (IQR: 12.3–15), respectively. By using the WHO classification, 9.48% (*n* = 29) of study participants were categorised as being malnourished, of whom 82.8% (24) had SAM (Table 1).

There were no significant differences between participants with or without malnutrition in terms of sex, age and birth weight. However, participants with employed fathers (*p* < 0.001) or mothers (*p* = 0.002), those breastfed (*p* < 0.01) and those from households with income > R2000 per month (*p* < 0.001) were significantly less likely to be admitted for any category of malnutrition. On the contrary, participants who presented with diarrhoea (*p* < 0.001) were significantly more likely to be admitted for malnutrition (Tables 4 and 5).

**TABLE 4:** Comparison of participants with and without malnutrition in terms of socio-demographic characteristics of parent or caregiver.

Socio-demographic characteristics	Malnutrition (MAM and SAM)					MAM					SAM				
	No		Yes		*p*	No		Yes		*p*	No		Yes		*p*
	*n*	%	*n*	%		*n*	%	*n*	%		*n*	%	*n*	%	
**Mother’s age (years)**															
Less than 18	4	1.4	0	-	0.727	4	1.4	0	0.0	0.601	4	1.4	0	0.0	0.677
18–35	231	83.4	26	89.7		231	83.4	4	80.0		231	83.4	22	91.7	
More than 35	42	15.2	3	10.3		42	15.2	1	20.0		42	15.2	2	8.4	
**Mother’s marital status**															
Single	183	66.1	19	65.5	0.953	183	66.1	4	80.0	0.666	183	66.1	15	62.5	0.823
Married or living together	0	0.0	0	0.0		0	0.0	0	0.0		0	0.0	0	0.0	
Divorced	0	0.0	0	0.0		0	0.0	0	0.0		0	0.0	0	0.0	
Widowed	94	33.9	10	34.5		94	33.9	1	20.0		94	33.9	9	37.5	
**Mother’s educational status**															
Never	22	7.9	3	10.3	0.691	22	7.9	1	20.0	0.520	22	7.9	2	8.3	0.850
Primary	28	10.1	3	72.4		28	10.1	0	80.0		28	10.1	3	12.5	
Secondary	186	67.2	21	10.3		186	67.2	4	0.0		186	67.2	17	70.8	
Tertiary	41	14.8	2	6.9		41	14.8	0	0.0		41	14.8	2	8.3	
**Father’s educational status**															
Never	87	31.4	8	27.6	0.401	87	31.4	2	40.0	1.000	87	31.4	6	25.0	0.602
Primary	0	0.0	0	0.0		0	0.0	0	0.0		0	0.0	0	0.0	
Secondary	150	54.2	19	65.5		150	54.2	3	60.0		150	54.2	16	66.7	
Tertiary	40	14.4	2	6.9		40	14.4	0	0.0		40	14.4	2	8.3	
**Mother’s employment status**															
No	190	68.6	28	96.6	0.002	190	68.6	5	100.0	0.328	190	68.6	23	95.8	0.004
Yes	87	31.4	1	3.5		87	31.4	0	0.0		87	31.4	1	4.2	
**Father’s employment status**															
No	100	36.1	22	75.9	< 0.001	100	36.1	5	100.0	0.007	100	36.1	17	70.8	0.002
Yes	177	63.9	7	24.1		177	63.9	0	0.0		177	63.9	7	29.2	
**Household income category**															
Less or equal to R2000.00	127	45.9	27	93.1	< 0.001	127	45.9	5	100.0	0.022	127	45.9	22	91.7	< 0.001
More than R2000.00	150	54.1	2	6.9		150	54.1	0	0.0		150	54.1	2	8.3	
**Number of dependants**															
Four or less dependants	211	76.2	26	89.7	0.098	211	76.2	5	100.0	0.594	211	76.2	21	87.5	0.310
More than four dependants	66	23.8	3	10.3		66	33.8	0	0.0		66	23.8	3	12.5	
**Source of water**															
Public tap	43	15.5	7	24.1	0.190	36	13.0	2	40.0	0.295	43	15.5	5	20.8	0.164
Private	198	71.5	16	55.2		198	71.5	3	60.0		198	71.5	13	54.2	
Open in the yard	36	13.0	6	20.7		43	15.5	0	0.0		36	13.0	6	25.0	
**Caregiver’s relationship with the child**															
Mother	256	92.4	27	93.1	0.660	256	92.4	4	80.0	0.206	256	92.4	23	95.8	0.710
Grandmother	5	1.4	1	3.5		5	1.8	1	20.0		5	1.8	0	0.0	
Caregiver	6	2.2	1	3.5		6	2.2	0	0.0		6	2.2	1	4.2	
Father	10	3.6	0	0.0		10	3.6	0	0.0		10	3.6	0	0.0	

MAM, moderate acute malnutrition; SAM, severe acute malnutrition.

**TABLE 5:** Comparison of participants with malnutrition to those without malnutrition in terms of health characteristics of children.

Health characteristics	Malnutrition (MAM and SAM)		MAM					SAM							
	No		Yes		*p*	No		Yes		*p*	No		Yes		*p*
	*n*	%	*n*	%		*n*	%	*n*	%		*n*	%	*n*	%	
**Concurrent illness**															
Diarrhoea	42	15.1	22	75.9	< 0.001	42	15.1	3	60.0	0.030	42	15.1	19	79.2	< 0.001
Pneumonia	155	56.0	2	6.9		155	56.0	2	40.0		155	56.0	3	12.5	
Other	80	28.9	5	17.2		80	28.9	0	0.0		80	28.9	2	8.3	
**Frequency of the diarrhoea**															
Rarely	20	44.4	10	55.6	0.796	20	44.4	1	33.3	0.739	20	44.4	9	60.0	0.501
Sometimes	19	42.2	6	33.3		19	42.2	2	66.7		19	42.2	4	26.7	
Very often	6	13.3	2	11.1		6	13.3	0	0.0		6	13.3	2	13.3	
**Children’s immunisation status up to date**															
No	6	2.2	2	6.9	0.170	6	2.2	0	0.0	0.739	6	2.2	2	8.3	0.127
Yes	271	97.8	27	93.1		271	97.8	5	100.0		271	97.8	22	91.7	
**Missed immunisation**															
Vitamin A	1	20.0	0	0.0	1.000	1	20.0	0	0.0	1.000	1	20.0	0	0.0	1.000
Deworming and vitamin A	1	20.0	0	0.0		1	20.0	0	0.0		1	20.0	0	0.0	
Oral polio vaccine 1	2	40.0	1	100.0		2	40.0	0	0.0		2	40.0	1	100.0	
Measles	1	20.0	0	0.0		1	20.0	0	0.0		1	20.0	0	0.0	
**Children ever been breastfed**															
No	70	26.1	18	66.7	< 0.001	70	26.1	2	50.0	0.286	70	26.1	16	69.6	< 0.001
Yes	198	73.9	9	33.3		198	73.9	2	50.0		198	73.9	7	30.4	

MAM, moderate acute malnutrition; SAM, severe acute malnutrition.

On tests of association, father’s employment status (*p* < 0.01), mother’s employment status (*p* = 0.02), household income (*p* < 0.01), child’s breastfeeding status (*p* < 0.01) and presenting medical problem (*p* < 0.01) were significantly associated with admission for malnutrition.

In the final multivariable regression analysis (Table 6), compared with others, participants who were not breastfed (odds ratio [OR] = 3.9; 95% CI: 1.23–12.29; *p* = 0.02) and those who presented with diarrhoea on admission (OR = 23.3; 95% CI: 6.85–79.43; *p* < 0.01) were significantly more likely to be admitted for any category of malnutrition. The strengths of these relationships were stronger for SAM only; compared with others, participants who were not breastfed (OR = 6.8; 95% CI: 1.93–23.89; *p* = 0.003) and those who presented with diarrhoea on admission (OR = 35.24; 95% CI: 8.10–153.25; *p* < 0.01) were significantly more likely to be admitted for SAM.

**TABLE 6:** Adjusted multivariable regression analysis of factors independently associated with malnutrition (moderate acute malnutrition and severe acute malnutrition).

Factors	Odds ratio	*p*	95% CI
**Household income**			
More than R2000	1	-	-
Less or equal to R2000	5.0	0.58	0.95–26.6
**Mother’s employment status**			
Employed	1	-	-
Unemployed	7.1	0.76	0.82–61.5
**Father’s employment status**			
Employed	1	-	-
Unemployed	2.3	0.167	0.70–7.8
**Breastfed**			
Breastfed	1	-	-
Never breastfed	3.9	0.021	1.23–12.29
**Concurrent illness**			
Pneumonia	1	-	-
Diarrhoea	23.3	< 0.001	6.85–79.43
Other	0.46	0.50	0.05–4.28

CI, confidence interval.

## Discussion

This study revealed a high prevalence of acute malnutrition (9.4%) with most children affected (82.8%) suffering from SAM. In addition, the feeding practices offered to the children in the study were poor and placed them at significant risk of malnutrition. Although household income and parents’ employment status appeared to influence malnutrition, having diarrhoea at presentation and a history of not being breastfed significantly increased the risk of malnutrition.

Although a prevalence of malnutrition of 26.0% has been reported in a study conducted in the same age group in Cape Town,^12^ a prevalence of 9.4% is still high and has serious clinical and public health implications. According to the DHIS in Sedibeng District, the case fatality rate is high (2.4%).^9^ Put together, both findings suggest that children at risk of malnutrition are not being identified early for intervention in primary care. Strategies that compel healthcare practitioners in primary care to screen for malnutrition such as including anthropometric measurements (weight, height/length and MUAC) as part of the vital signs during every clinic visit by a child are needed to improve early detection of malnutrition. Those found to be at risk should be immediately referred to a dietician for appropriate interventions and linked with the ward-based outreach team for follow-up at the community level.

That 70.2% of children in this study were breastfed at all is a welcome finding and may reflect the strength of the campaign on exclusive breastfeeding by the South African Department of Health.^12^ However, only 57.4% were breastfed for at least 6 months – the commonest reasons for stopping early being sickness of the mother or the baby (32.1%) and breast problem (26.2%). Considering that 71.2% of mothers were unemployed, mothers ought to have had enough time for exclusive breastfeeding for 6 months. To compound the problem of poor nutritional practices, 19.05% of children were started on solid foods before 6 months of age. The later practice has been reported to have detrimental effects on the health of infants.^13^ The feeding practices found in this study therefore suggest the need to intensify dietary education and promotion of breastfeeding during antenatal care visits, more so, that a history of not being breastfed significantly increased the risk of malnutrition in this study.

Most participants had three meals on the average per day and, at best, an additional snack per day. This is not only inadequate to support normal childhood development; it falls far short of the daily requirement of six to eight meals recommended for the study population.^14^ Although this could be because of unavailability of food and poor knowledge on diet, it raises serious concerns considering that inadequate food intake is a risk for malnutrition that, in turn, predispose to physical and cognitive underdevelopment and poor quality of life.^14^ In the first 6 months of life, promoting exclusive breastfeeding given on demand or at least every 2 h and the provision of dietary education have the potential to increase the number of meals per day found in this study. Beyond 6 months, dietary education at the clinical coalface and empowerment of community health workers to teach carers the correct feeding practice are interventions that could address the small number of meals. At systems level, inter-sectorial collaboration across government departments may address socio-economic depravities and promote access and judicious use of social grants, food banks and female self-reliance through small-scale businesses.

The relationship between poor household income (< R2000 per month) and malnutrition in this study reiterates findings of a previous study that showed parental unemployment linked to poor household income as a risk factor for malnutrition.^15^ In essence, although screening can identify children at risk for nutritional and clinical interventions, the underlying problem in many cases is socio-economic, and it may therefore be more appropriate to address the socio-economic depravities that place these children at risk of malnutrition through inter-sectorial collaboration highlighted above. This study found that both parents being employed protected against malnutrition and that mothers’ employment did so to a greater extent than the fathers’ (Tables 5 and 6) – suggesting a need for further studies to examine why this disparity in effect exists.

The strong association between diarrhoea and malnutrition (OR = 23.3; 95% CI: 6.85–79.43; *p* < 0.01) in this study is consistent with findings of other studies^16,17^ and indicates the importance of prompts that compel clinicians to do anthropometric measurements in children who present with diarrhoea.

This study reports a very high immunisation coverage, including Rota virus, that has been associated with significant reduction in the incidence of diarrhoea.^17^ On the contrary, malnutrition may reduce the effectiveness of Rota virus.^18,19^ Although intuitive, it is not clear whether the introduction of Rota virus immunisation has led to any reduction in the prevalence of malnutrition in South Africa.

This was a cross-sectional study and the associations found were only suggestive and not causal in nature. Considering that several study measures relied on self-reports, there is a potential for recall and information bias, with possible misclassifications. Intra-performer variability during anthropometric measurements could also have resulted in a margin of human error that might have affected some study estimates. However, the same researcher performed the anthropometric measurements and adhered strictly to the WHO guidelines. Notwithstanding these potential limitations, the findings of this multicentre study provide an understanding of malnutrition and its associated factors within the context of a typical South African peri-urban health district that are useful for informing interventions in this or similar settings.

## Conclusion

A significant proportion of participants had malnutrition and was subjected to suboptimal feeding practices. Healthcare providers in primary care need to entrench dietary education and anthropometric screening in all clinic visits for children < 5 years old, particularly when they present with diarrhoea or are not being breastfed.
